# Association between cardiometabolic index and controlled attenuation parameter in U.S. adults with NAFLD: findings from NHANES (2017–2020)

**DOI:** 10.1186/s12944-024-02027-x

**Published:** 2024-02-07

**Authors:** Wen-feng Xi, Ai-ming Yang

**Affiliations:** Department of Gastroenterology, State Key Laboratory of Complex Severe and Rare Diseases, Peking Union Medical College, Peking Union Medical College Hospital, Chinese Academy of Medical Sciences, Beijing, China

**Keywords:** Non-alcoholic fatty liver disease, CMI, NHANES, Crosssectional study

## Abstract

**Background:**

Cardiometabolic index (CMI), a novel indicator that combines abdominal obesity and lipid levels, has been confirmed to correlate with non-alcoholic fatty liver disease (NAFLD). However, limited research has been conducted on the relationship between CMI and controlled attenuation parameter (CAP), a parameter measured by transient elastography and reflecting the extent of fat accumulation in the liver. The objective of our study was to investigate the relationship between the two variables.

**Methods:**

This was a cross-sectional study with a sample size of 1,759 U.S. adults with NAFLD sourced from the NHANES 2017–2020. Participants with a median CAP ≥ 248 dB/m were considered to have hepatic steatosis. CMI was calculated as [waist circumference (cm)/height(cm)]×[TG (mmol/L)/HDL-C (mmol/L)]. Multivariate linear regression, generalized additive model and subgroup analysis were employed to examine the association of CMI and CAP.

**Results:**

The average age of the 1,759 participants was 50.2 years, with males accounting for 50.76% and females 49.24%. The average BMI was 32.23 kg/m². The multivariate linear regression model indicated that with every 1-unit increase in CMI, there was an associated rise of 10.40 dB/m in CAP (95% CI, 7.14–13.67) after adjusting for covariates and a *p* for trend < 0.05 suggested the existence of a linear association between the two variables. Similarly, generalized additive model also found it a roughly linear relationship between the two. Subgroup analysis revealed a positive correlation in the majority of subgroups.

**Conclusions:**

CMI was positively associated with CAP in U.S. adults with NAFLD. Our findings indicated that CMI may serve as an ideal indicator for monitoring the degree of hepatic steatosis among patients with NAFLD.

**Supplementary Information:**

The online version contains supplementary material available at 10.1186/s12944-024-02027-x.

## Introduction

Non-alcoholic fatty liver disease (NAFLD) stands as the predominant etiological factor behind chronic liver diseases with a prevalence of up to approximately 25% [1]. Concurrently, with the escalating prevalence of obesity and metabolic syndrome, the global incidence of NAFLD exhibits an upward trajectory. NAFLD harbors the potential for deleterious progression, ranging from non-alcoholic steatohepatitis (NASH) to hepatocellular carcinoma [2]. As a hepatic manifestation of metabolic syndrome, NAFLD interlinks with an array of metabolic disorders such as type 2 diabetes, cardiovascular diseases and hyperuricemia [3]. Given its high prevalence and intricate associations with other diseases, it is evident that NAFLD warrants urgent attention as a pressing public health concern.

Transient elastography (TE) is a non-invasive and convenient ultrasonography technique for assessing the degree of hepatic fibrosis and steatosis in patients with liver disease and is now widely in use. It encompasses two primary parameters, namely liver stiffness measurement (LSM) and controlled attenuation parameter (CAP). LSM quantifies the degree of liver fibrosis, providing a numeric value that correlates with the stiffness of the hepatic tissue while CAP focuses on assessing hepatic steatosis, quantifying the amount of fat in the liver. To a certain extent, it serves as an ideal alternative to liver biopsy [4].

Some indices have been found to be associated with metabolism-related diseases, such as body mass index (BMI) [5], waist-to-hip ratio (WHR) [6], waist-to-height ratio (WHtR) [7], the ratio of TG and HDL-C (TG/HDL-C) [8] and so forth. CMI, proposed by Japanese scholar Wakabayashi in 2015 and calculated by multiplying TG/HDL-C ratio and WHtR, is nowadays regarded as a novel estimate of visceral adipose tissue [9, 10]. And there have been researches linking this index to type 2 diabetes [9], stroke [11], the degree of atherosclerosis in peripheral arterial disease [12] and left ventricular geometry change [13].

Although it has been shown that CMI is associated with the likelihood of developing NAFLD [14, 15], limited information is available regarding the relationship between CMI and CAP. Exploring the relationship between the two may offer novel insights for monitoring the severity of NAFLD, requiring only lipid profiles and body measurements. CMI could be a convenient and cost-effective indicator for assessing the disease. Accordingly, we conducted the present study.

## Methods

### Study design and participants

This was a cross-sectional study with data from the National Health and Nutrition Examination Survey (NHANES), which is administered by the Centers for Disease Control and Prevention (CDC) and began in the early 1960s and is still ongoing with the aim of assessing the health and nutritional status of the U.S. people. The NHANES contents include demographics data, questionnaire information, examination data laboratory data and other information. In the current study, the NHANES data from 2017 to 2020 were utilized as this is the only period that included information about TE of the current moment.

**Fig. 1:**
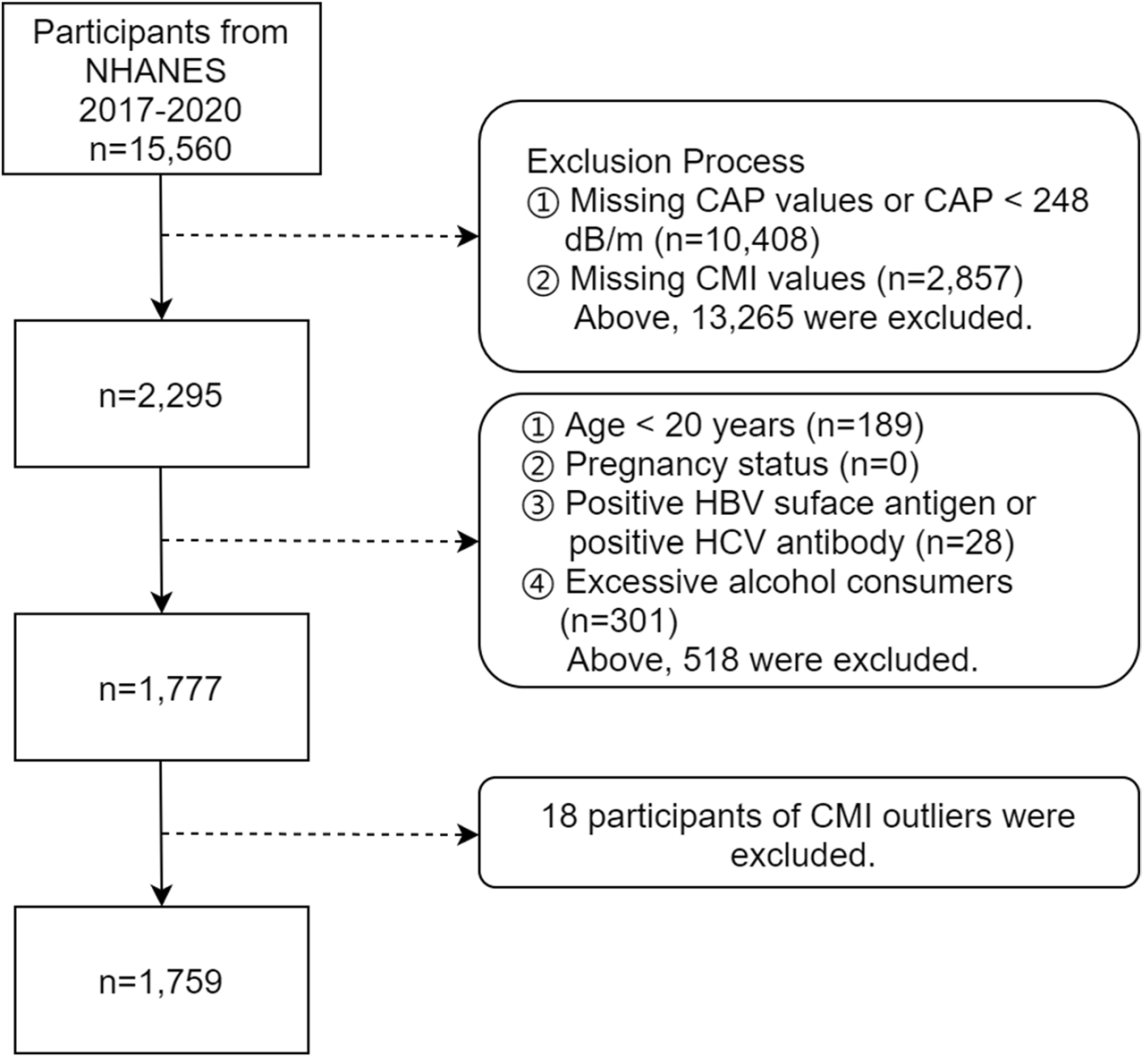
Flow chart for inclusion of participants in this study

Figure 1 illustrates the participants exclusion process. Specific exclusion criteria were applied as follows: (1) Missing CAP values or CAP < 248 dB/m, (2) Missing CMI values, (3) Age less than 20 years, (4) Pregnancy status, (5) Positive HBV surface antigen or positive HCV antibody, (6) Excessive alcohol consumers (ever have 4/5 or more drinks every day). After excluding cases meeting the aforementioned conditions, a total of 1,777 participants remained and an initial statistical analysis was performed on the sample of 1,777, wherein outliers in CMI were found. Following the criterion of considering CMI values exceeding the 99th percentile as outliers, 18 participants were further excluded. Ultimately, the analysis included a final sample of 1,759.

NHANES study protocols were approved by the Research Ethics Review Board of the National Center for Health Statistics. Written informed consent was obtained from all participants in this study. More information regarding NHANES can be found at the official website [16].

### CMI

CMI = [WC (cm)/height(cm)]×[TG (mmol/L)/HDL-C (mmol/L)] [9].

CMI was considered the exposure variable.

### Liver steatosis and CAP

CAP, measured by TE, indicates the degree of hepatic steatosis, with higher values corresponding to more significant hepatic steatosis. In the current study, median CAP of 248dB/m was deemed as the best cut-off value for the diagnosis of hepatic steatosis [17]. CAP value was treated as the outcome variable.

### Covariates

Covariates included age (year), gender (male or female), race (Non-Hispanic White or Non-Hispanic Black or Mexican American or other), education level (< high school or ≥ high school), marital status (Married or living with partner or never married or other), income to poverty ratio (PIR), smoking (yes or no), moderate work activity (yes or no), body mass index (BMI, kg/m2), systolic blood pressure (SBP, mmHg), diastolic blood pressure (DBP, mmHg), low-density lipoprotein-cholesterol (LDL-C, mmol/L), alanine transaminase (ALT, U/L), aspartate transaminase (AST, U/L), gamma glutamyl transferase (GGT, IU/L), albumin (ALB, g/L), UA (uric acid, mg/dL), hemoglobin A1c (HbA1c, %), liver stiffness measurement (LSM, kPa), diabetes (yes or no), hypertension (yes or no), hyperuricemia (yes or no), stroke (yes or no), etc. In the subgroup analysis, age was categorized as < 45, 45–65 and ≥ 65 years, BMI as < 25, 25–30 and ≥ 30 kg/m^2^ and ALT as ≤ 40 and > 40U/L .

### Statistical analysis

Continuous variables were expressed as mean ± standard deviation (SD) and categorical variables as proportions. CMI was divided into tertiles from the lowest (T1) to the highest (T3). The differences among participants grouped by CMI tertiles were assessed using a weighted linear regression model (continuous variables) or weighted chi-square test (categorical variables). Three linear regression models were used to explore the relationship between CMI and CAP. In model 1, no covariate was adjusted; in model 2, gender (with male as the reference), age and race (with Non-Hispanic White as the reference) were adjusted; model 3 further adjusted for education level (with <High school as the reference), marital status (with Married/Living with Partner as the reference), PIR, moderate work activity (with Yes as the reference), BMI, SBP, DBP, ALT, AST, GGT, ALB, LDL-C, uric acid, LSM, smoking (with Yes as the reference), diabetes (with Yes as the reference), stroke (with Yes as the reference) based on model 2. Variance inflation factors were calculated, and Spearman’s correlation analysis was employed to examine collinearity among variables. Trend test was performed across CMI tertile groups. β coefficients (β) and 95% confidence intervals (95% CIs) were reported. A generalized additive model (GAM) was applied to find whether CMI and CAP were in a non-linear relationship. Piece-wise regression was used for dividing CMI into segments and fitting a separate regression model to each segment and we employed the log-likelihood ratio test to assess which model provided a better fit. Subgroup analysis was conducted to assess the robustness of the results and interaction test was performed. Participants were divided into different subgroups based on gender, age, BMI, ALT, and specific diseases and these stratified factors were also regarded as pre-specified potential effect modifiers. Data were analyzed with the use of R version 4.2.0 (http://www.r-project.org, The R Foundation) and Empower software (www.empowerstats.com, X&Y solutions, inc. Boston, Massachusetts). Appropriate NHANES sampling weights were used in the statistical analysis. The significance level was set at *p*<0.05.

## Results

### Characteristics of participants

Finally, the study comprised a total of 1,759 participants aged ≥ 20 years, with males accounting for 50.76% and female 49.24% and the mean age was 50.19 years. CMI was divided into tertiles: the ranges were 0.05–0.45, 0.45–0.85, 0.85–3.90, respectively. And the participants were divided into three groups based on CMI tertiles.

Among tertiles, there were significant differences in the distribution of gender, race, education level, moderate work activity, diabetes, hypertension and hyperuricemia (all *p*<0.05). Compared to the other two groups, participants in tertile 3 displayed a propensity for larger waist circumference, higher BMI and a higher prevalence of diabetes, hypertension, hyperuricemia and stroke. Of utmost significance, participants in tertile 3 demonstrated higher value for CAP. Detailed information is presented in Table 1.

**Table 1:** Weighted baseline characteristics of study participants according to tertile groups of CMI

	Overall (0.05–3.90)	T1 (0.05–0.45)	T2 (0.45–0.85)	T3 (0.85–3.90)	* p* value
No. of participants	1759	586	586	587	
Age (years)	50.2 ± 16.2	50.4 ± 17.0	50.7 ± 15.6	49.4 ± 15.9	0.3605
Gender (%)					0.0024
Male	50.76	49.33	46.24	56.19	
Female	49.24	50.67	53.76	43.81	
Race (%)					< 0.0001
Non-Hispanic White	63.96	66.03	57.69	67.44	
Non-Hispanic Black	8.82	11.69	10.90	4.08	
Mexican American	10.11	8.20	9.99	12.14	
Other	17.10	14.08	21.41	16.34	
Education level (%)					0.0010
<High school	10.76	7.11	11.96	13.40	
≥High school	89.24	92.89	88.04	86.60	
Marital status (%)					0.7860
Married/Living with Partner	69.22	67.94	69.41	70.34	
Never married	14.16	14.64	13.31	14.42	
Other	16.63	17.43	17.27	15.24	
PIR	3.17 ± 1.60	3.36 ± 1.56	3.11 ± 1.67	3.03 ± 1.57	0.0025
Smoking (%)					0.1126
Yes	42.00	40.39	40.01	45.38	
No	58.00	59.61	59.99	54.62	
Moderate work activity (%)					< 0.0001
Yes	49.67	55.97	41.92	50.16	
No	50.33	44.03	58.08	49.84	
Waist circumference (cm)	107.19 ± 15.73	100.65 ± 14.51	107.88 ± 14.45	113.18 ± 15.48	< 0.0001
BMI (kg/m^2^)	32.23 ± 7.19	29.44 ± 6.14	32.62 ± 6.88	34.70 ± 7.45	< 0.0001
SBP (mmHg)	123.11 ± 16.60	122.32 ± 16.79	123.14 ± 17.22	123.88 ± 15.82	0.2866
DBP (mmHg)	76.04 ± 11.13	74.27 ± 10.79	76.60 ± 10.73	77.33 ± 11.56	< 0.0001
TG (mmol/L)	1.39 ± 0.76	0.75 ± 0.24	1.25 ± 0.31	2.17 ± 0.71	< 0.0001
LDL-C (mmol/L)	2.90 ± 0.93	2.77 ± 0.81	2.99 ± 1.00	2.96 ± 0.95	< 0.0001
HDL-C (mmol/L)	1.32 ± 0.38	1.61 ± 0.41	1.28 ± 0.24	1.05 ± 0.18	< 0.0001
ALT (U/L)	24.66 ± 16.95	20.95 ± 13.79	25.36 ± 16.59	27.80 ± 19.27	< 0.0001
AST (U/L)	21.80 ± 11.09	21.24 ± 11.85	21.97 ± 10.96	22.21 ± 10.36	0.2918
GGT (IU/L)	31.38 ± 33.11	28.24 ± 35.31	30.17 ± 28.19	35.61 ± 34.37	0.0003
ALB (g/L)	40.25 ± 3.10	40.50 ± 3.01	40.22 ± 3.05	40.01 ± 3.22	0.0243
Uric acid (mg/dL)	5.66 ± 1.35	5.26 ± 1.20	5.75 ± 1.37	5.98 ± 1.38	< 0.0001
HbA1c (%)	5.82 ± 1.05	5.51 ± 0.65	5.86 ± 0.95	6.10 ± 1.33	< 0.0001
eGFR(ml/min/1.73m^2^)	95.56 ± 25.76	92.98 ± 21.87	96.49 ± 27.10	97.34 ± 27.89	0.0077
LSM (kPa)	6.16 ± 4.39	5.46 ± 2.85	5.92 ± 3.11	7.09 ± 6.13	< 0.0001
CAP (dB/m)	305.76 ± 41.11	290.07 ± 35.39	305.38 ± 39.05	321.95 ± 42.02	< 0.0001
Diabetes (%)					< 0.0001
Yes	14.71	6.61	15.15	22.50	
No	85.29	93.39	84.85	77.50	
Hypertension (%)					< 0.0001
Yes	47.84	37.94	52.53	53.79	
No	52.16	62.06	47.47	46.21	
Hyperuricemia (%)					< 0.0001
Yes	26.11	14.81	28.89	35.07	
No	73.89	85.19	71.11	64.93	
Stroke (%)					0.3420
Yes	3.31	3.46	2.41	3.94	
No	96.69	96.54	97.59	96.06	

For continuous variables, variables are presented as mean ± standard deviation and *p* value was calculated by weighted linear regression model. For categorical variables, variables are displayed as percentage and *p* value was calculated by weighted chi-square test

*PIR* Income to poverty ratio, *BMI* Body mass index, *SBP* Systolic blood pressure, *DBP* Diastolic blood pressure, *TG* Triglyceride, *LDL-C* Low density lipoprotein cholesterol, *HDL-C* High density lipoprotein cholesterol, *ALT* Alanine transaminase, *AST* Aspartate transaminase, *GGT* Gamma glutamyl transferase, *ALB* Albumin, *HbA1c* Hemoglobin A1c, *eGFR* Estimated glomerular filtration rate, *LSM* Liver stiffness measurement, *CAP* Controlled attenuation parameter

### The association between CMI and CAP

Three linear regression models were applied to investigate the association between CMI and CAP and the number of adjusted covariates varied across the three models, ranging from unadjusted (Model 1) to fully adjusted (Model 3). Table 2 presents the aforementioned information in detail. In terms of collinearity, the variance inflation factors for the variables are all below 5 (eTable 1), and the |r| (Spearman correlation analysis) is less than 0.8 (eFig 1), indicating the absence of severe collinearity.

**Table 2:** Linear regression analysis between CMI and CAP

Exposure	Model 1 β (95%CI)	Model 2 β (95%CI)	Model 3 β (95%CI)
CMI value (per 1 unit increase)	22.78 (19.66, 25.91)***	22.97 (19.82, 26.13)***	10.40 (7.14, 13.67)***
Tertiles of CMI			
T1	0	0	0
T2	15.31 (10.81, 19.82)***	15.60 (11.08, 20.13) ***	4.59 (-0.01, 9.19)
T3	31.89 (27.52, 36.25)***	31.96 (27.55, 36.36) ***	13.11 (8.43, 17.79) ***
*p* for trend	< 0.0001	< 0.0001	< 0.0001

Model 1 unadjusted

Model 2 adjusted for age, gender and race

Model 3 further adjusted for education level, marital status, smoking, moderate work activity, PIR, SBP, DBP, BMI, LSM, UA, LDL-C, ALT, AST, ALB, GGT, diabetes and stroke based on Model 2

*CMI* Cardiometabolic index, *T* Tertile

***p<0.001

**Fig. 2:**
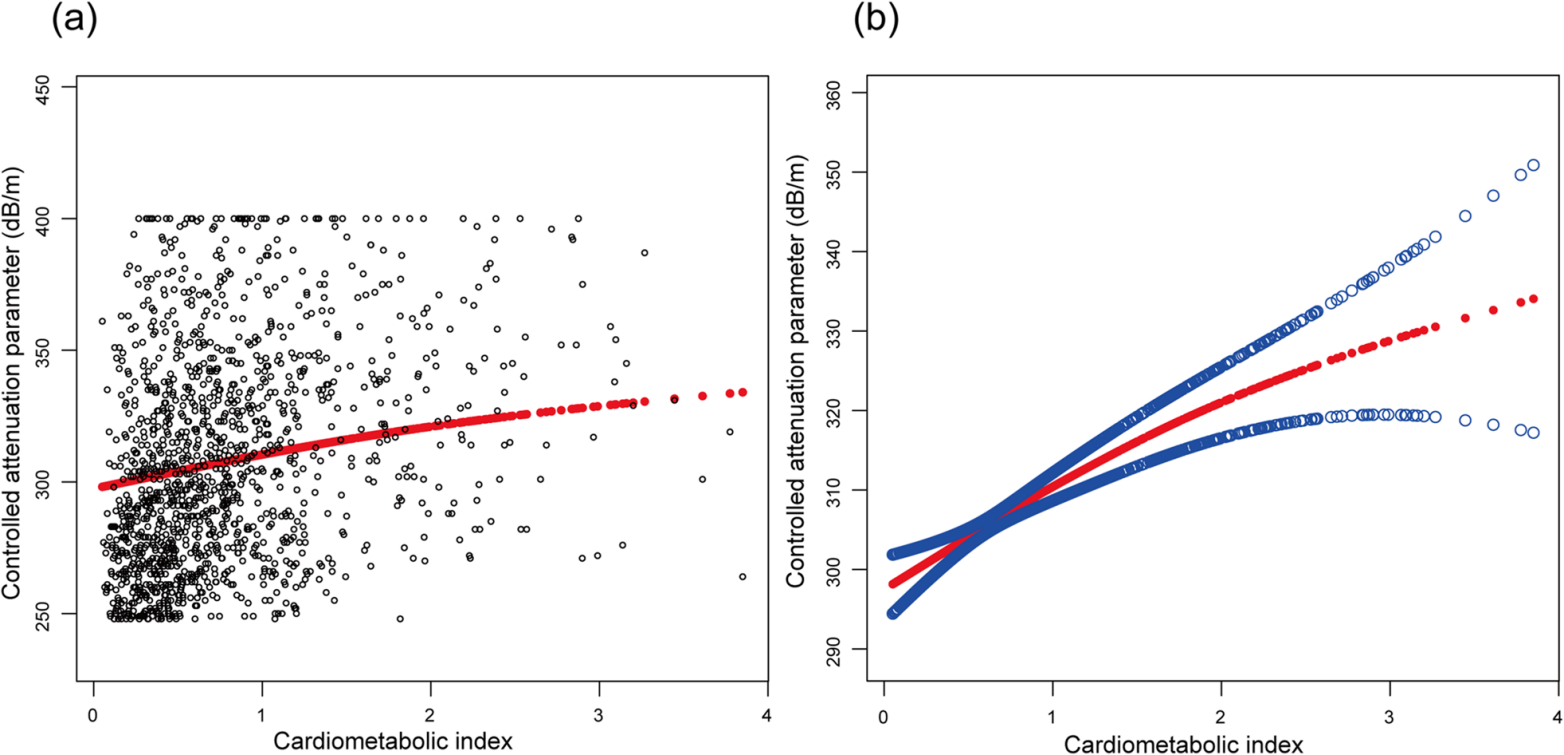
The association between cardiometabolic index and controlled attenuation parameter. (**a**) Scatter plot, where each black point represents a sample. (**b**) Smooth curve fitted by a generalized additive model. The red line denoted the fitted curve and the two blue lines represented 95% confidence intervals. Model adjusted for gender, race, age, education level, marital status, PIR, moderate work activity, smoking, SBP, DBP, BMI, LDL-C, ALT, AST, GGT, ALB, UA, LSM, diabetes and stroke

A positive association between CMI and CAP was found in all three models (Model 1: β, 22.78; 95% CI, 19.66–25.91; Model 2: β, 22.97; 95% CI, 19.82–26.13; Model 3: β, 10.40; 95% CI, 7.14–13.67). The difference in β between Models 2 and 3 was large. Therefore we established 18 linear regression equations (including Model 2 and 3), introducing one new variable at a time and reported the change in the coefficient of determination (∆R²) (eTable 2). The results indicated that BMI played a predominant role in the variation of β. After stratifying the CMI into tertiles (Tertile 1–3), trend test was conducted and a linear relationship between CMI and CAP was indicated by all three models (all *p* for trend<0.05). Furthermore, generalized additive model and smooth curve fitting were employed to investigate possible non-linear relationship and the result also supported a positive linear relationship as shown in Fig. 2. The linear relationship between the two was reinforced by two-piecewise regression (*p* for log-likelihood ratio > 0.05, the linear regression providing a better fit), as indicated in Table 3.

**Table 3:** Association between CMI and CAP using two-piecewise linear regression

	Adjusted β (95% CI), *p* value
Model 1 (linear regression)	10.40 (7.14, 13.67), < 0.0001
Model 2 (segmented regression)	
Turning point(K)^a^	1.43
CMI < K effect	14.58 (9.03, 20.14), < 0.0001
CMI > K effect	3.74 (−4.13, 11.61), 0.3519
* p* for log-likelihood ratio	0.066

Fully adjusted model: age, gender, race, education level, marital status, smoking, moderate work activity, PIR, SBP, DBP, BMI, LSM, UA, LDL-C, ALT, AST, ALB, GGT, diabetes and stroke

*CMI* Cardiometabolic index

^a^Two-step recursive method was employed to determine the inflection point

### Subgroup analysis

**Fig. 3:**
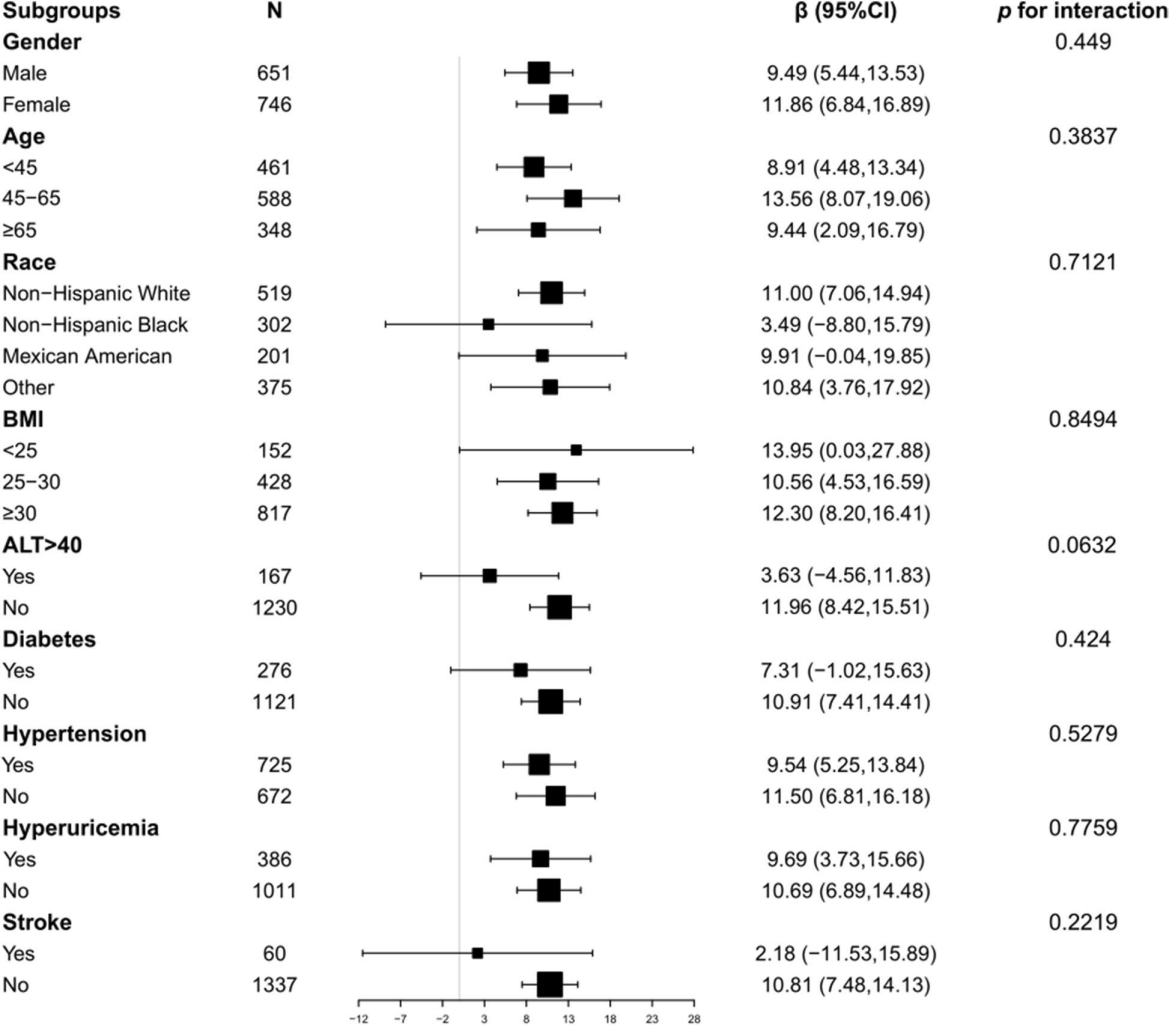
Subgroup analysis for the association between CMI and CAP. Covariates to be adjusted included gender, race, age, education level, marital status, PIR, moderate work activity, smoking, SBP, DBP, BMI, LDL-C, ALT, AST, GGT, ALB, UA, LSM, diabetes and stroke and covariates related to stratification factors were not adjusted

Participants were stratified into distinct subgroups based on gender, age, race, BMI, ALT, diabetes, hypertension, hyperuricemia, and stroke status. After adjustment for confounding factors, β coefficients of CMI on CAP remained consistent across all subgroups (all β>0), notwithstanding that this positive correlation exhibits some degree of instability in certain subgroups, including Non-Hispanic Blacks, Mexican Americans, group with BMI < 25, group with ALT > 40, diabetic patients, and stroke patients (95% CI lower limit < 0 or broader 95% CI). Details are shown in Fig. 3.

## Discussion

In our cross-sectional study including 1,759 U.S. adults with NAFLD, the positive association between CMI and CAP was found. Serving as sensitivity analysis, a generalized additive model and subgroup analysis further confirmed the robustness of the positive association.

CMI is the product of TG/HDL-C and WHtR, combining lipid levels and visceral obesity [9]. Studies showed that TG/HDL-C is an efficient predictor of cardiovascular disease [18], and WHtR, i.e. waist circumference corrected by height, is a good indicator of abdominal obesity and considered as a superior tool to BMI and waist circumference in screening for adult cardiometabolic risk factors [19]. Both TG/HDL and WHtR are correlated with insulin resistance and NAFLD [20–23]. As the integration of both, CMI should theoretically function as a strong indicator for assessing NAFLD.

We reviewed previous studies examining the relationship between CMI and NAFLD. Zou et al. [15] conducted post hoc analysis of NAGALA with a sample size of 14,251 subjects and revealed that CMI served as an independent risk factor for NAFLD. For each increase of 1 SD in CMI, the risk of NAFLD escalated by 28% (95% CI: 1.19–1.37). Furthermore, disparities were observed in the risk of NAFLD across different age and gender groups: with each increment of 1 SD in CMI, the risk was higher among the young people than the middle-aged and elderly people, and among women than men. Similar results were obtained in the study conducted by Liu et al [14]. In Liu’s study, CMI was divided into quartile groups and the prevalence of NAFLD increased in sequence from Q1 to Q4. Multivariate logistic regression suggested that the prevalence of NAFLD increased for each 1 SD elevation in CMI in both sexes (Male: OR, 3.069; 95% CI, 2.603–3.618; Female: OR, 3.110; 95% CI, 2.579–3.750). It is worth noting that the outcome variable in the aforementioned two studies, namely NAFLD, was a dichotomous categorical variable, and the primary emphasis of these two studies was directed towards investigating the correlation between CMI and the prevalence of NAFLD. While, the outcome variable in our study was CAP, a continuous variable assessing the degree of hepatic steatosis. Correspondingly, multivariate analysis employed a linear regression model and the main emphasis of our study was to explore the quantitative variations in the degree of hepatic steatosis in adult NAFLD patients in relation to shifting levels of CMI. Accordingly, our study could be regarded as an extension and complement to the previous researches.

In addition to NAFLD, CMI is associated with a variety of diseases. Wakabayashi et al. [9] discovered that the correlations between CMI and hyperglycemia as well as diabetes are significant, establishing CMI as a helpful indicator for discriminating diabetes. In a later study, Wakabayashi [24] observed that CMI value in different age groups differed among genders: CMI were higher in the middle-aged men than in the youngest and the oldest while in women CMI rose with increasing age. Moreover, he found that the relationship between CMI and diabetes weakened with age. Liu et al. [25] found a stronger correlation between CMI and hyperuricaemia compared to body adiposity index (BAI), conicity index (CI), a body shape index (ABSI), body roundness index (BRI) and visceral adiposity index (VAI). With hyperuricaemia as the outcome variable, the OR in the highest quartile of CMI was 4.332 (95% CI, 3.938–4.765) with the lowest quartile as the reference. Wang et al. [11] identified the association between CMI and ischemic stroke: for every 1 SD increase in CMI, the risk of ischemic stroke increased by 18% (95% CI, 1.056–1.316) in women and 14% (95% CI, 1.016–1.270) in men.

### Study strengths and limitations

There were several advantages of this study. First, this study adjusted for potential covariates to mitigate the influence of confounding factors, thereby elucidating the independent relationship between CMI and CAP. Second, in addition to multivariate regression analysis, this study employed generalized additive models and subgroup analysis to ascertain the stability of the results. Third, transient elastography provided an objective means to diagnose hepatic steatosis thereby largely avoiding recall bias from self-reporting. Inevitably, there were limitations. To start with, due to the nature of the cross-sectional study design, it was not possible to establish a causal association between CMI and CAP. Next, the participants in this study were all U.S. adults, considering the influence of factors such as geography, genetics and culture, the applicability of the results of the present study to other geographic and racial groups might be constrained. Moreover, although some covariates were adjusted, there were still potential cofounding factors unadjusted. At last, the diagnosis of hepatic steatosis in this study relied on transient elastography rather than liver biopsy, which is widely regarded as the definitive method for identifying fatty liver disease.

## Conclusions

The study found that CMI was positively associated with CAP in U.S. adults with NAFLD and CMI may serve as an ideal indicator for monitoring the degree of hepatic steatosis among patients with NAFLD. Further large-scale prospective studies required to authenticate our findings.

### Supplementary Information

**Additional file 1.**

## Data Availability

The data in the current study can be found on the website: https://www.cdc.gov/nchs/nhanes/.

## Abbreviations

CMI: Cardiometabolic index
NAFLD: Non-alcoholic fatty liver disease
TE: Transient elastography
CAP: Controlled attenuation parameter
NHANES: National Health and Nutrition Examination Survey
PIR: Income to poverty ratio
BMI: Body mass index
SBP: Systolic blood pressure
DBP: Diastolic blood pressure
LDL-C: Low-density lipoprotein cholesterol
ALT: Alanine transaminase
AST: Aspartate transaminase
GGT: Gamma glutamyl transferase
ALB: Albumin
UA: Uric acid
HbA1c: Glycohemoglobin
LSM: Liver stiffness measurement
