# Supplementary material for: Association between cardiometabolic index and controlled attenuation parameter in U.S. adults with NAFLD: findings from NHANES (2017–2020)
Source: Lipids Health Dis. 2024 Feb 7;23:40. doi: 10.1186/s12944-024-02027-x (PMC10851511; doi:10.1186/s12944-024-02027-x)
Supplement: Supplementary file 1 — Additional file 1. [file 12944_2024_2027_MOESM1_ESM.docx]

Supplementary Materials

eFig 1.Heatmap of Spearman rank correlation coefficients

eTable 1. Variance inflation factors for variables

eTable 2. 18 linear regression equations with a new variable included at each step and △R^2^ reported

eTable 1. Variance inflation factors for variables

| Variables | VIF |
| --- | --- |
| Gender | 1.5 |
| Age | 1.7 |
| Race | 1.2 |
| CMI | 1.2 |
| Education level | 1.1 |
| Marital status | 1.2 |
| PIR | 1.4 |
| Smoking | 1.1 |
| Moderate work activity | 1.1 |
| SBP | 2.3 |
| DBP | 2 |
| BMI | 1.7 |
| LDL-C | 1.1 |
| ALT | 3.1 |
| AST | 2.9 |
| Albumin | 1.4 |
| GGT | 1.5 |
| Uric acid | 1.3 |
| LSM | 1.1 |
| Diabetes | 1.2 |
| Stroke | 1.1 |

eTable 2. 18 linear regression equations with a new variable included at each step and △R^2^ reported

| Step | Variables | β coefficient | R^2^ | △R^2^ |
| --- | --- | --- | --- | --- |
| 1 | Model 2 | 22.97275 | 0.1076 | - |
| 2 | Step 1 + education level | 23.34765 | 0.1112 | 0.0036 |
| 3 | Step 2 + marital status | 23.28219 | 0.1173 | 0.0061 |
| 4 | Step 3 + smoking | 23.35548 | 0.1185 | 0.0012 |
| 5 | Step 4 + moderate work activity | 23.23115 | 0.1210 | 0.0025 |

eTable 2. *(Continued)*

| Step | Variables | β coefficient | R^2^ | △R^2^ |
| --- | --- | --- | --- | --- |
| 6 | Step 5 + PIR | 22.76441 | 0.1251 | 0.0041 |
| 7 | Step 6 + SBP | 21.20292 | 0.1286 | 0.0035 |
| 8 | Step 7 + DBP | 20.61468 | 0.1342 | 0.0056 |
| 9 | Step 8 + BMI | 12.89873 | 0.2846 | 0.1504 |
| 10 | Step 9 + LSM | 12.70378 | 0.2984 | 0.0138 |
| 11 | Step 10 + UA | 12.48817 | 0.2987 | 0.0003 |
| 12 | Step 11 + LDL-C | 12.79004 | 0.3000 | 0.0013 |
| 13 | Step 12 + ALT | 11.95020 | 0.3178 | 0.0178 |
| 14 | Step 13 + AST | 11.67842 | 0.3200 | 0.0022 |
| 15 | Step 14 + Alb | 11.63611 | 0.3204 | 0.0004 |
| 16 | Step 15 + GGT | 11.65487 | 0.3204 | 0 |
| 17 | Step 16 + diabetes | 10.64141 | 0.3311 | 0.0107 |
| 18 | Step 17 + stroke | 10.40177 | 0.3333 | 0.0022 |

Model 2 adjusted for age, gender and race.


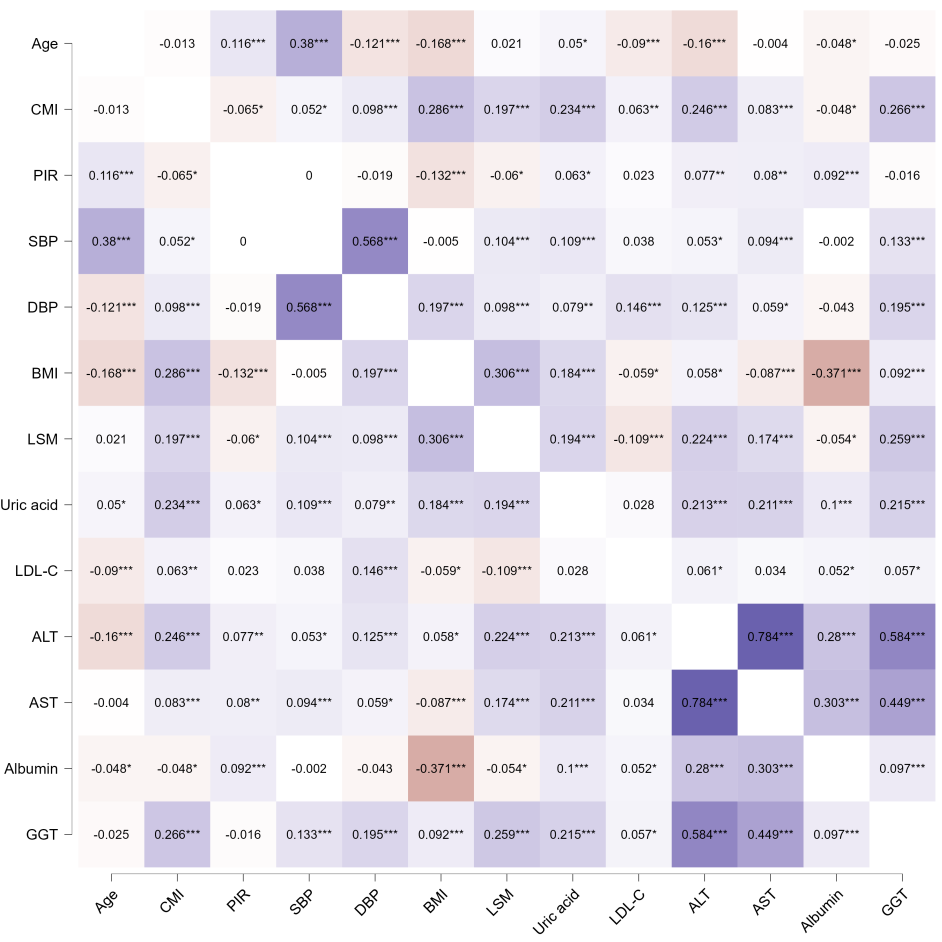


eFigure 1.Heatmap of Spearman’s rank correlation coefficients
